# Prehabilitation and Nutritional Support to Improve Perioperative Outcomes

**DOI:** 10.1007/s40140-017-0245-2

**Published:** 2017-11-07

**Authors:** Malcolm A. West, Paul E. Wischmeyer, Michael P. W. Grocott

**Affiliations:** 1 Academic Unit of Cancer Sciences, Faculty of Medicine, University of Southampton, Southampton, UK; 2 University Hospital Southampton NHS Foundation Trust, Southampton, UK; 3 University of Southampton NIHR Biomedical Research Centre, Southampton, UK; 4 Integrative Physiology and Critical Illness Group, Clinical and Experimental Sciences, Faculty of Medicine, University of Southampton, Southampton, UK; 5 Department of Anesthesiology, Duke University School of Medicine, Durham, NC USA; 6 Duke Clinical Research Institute, Durham, NC USA; 7 Department of Anaesthesia and Critical Care, University Hospital Southampton NHS Foundation Trust, Southampton, UK

**Keywords:** Multimodal prehabilitation, Nutrition, Exercise, Stress of surgery, Postoperative outcome, Mortality

## Abstract

**Purpose of Review:**

The purpose of this study is to evaluate the role of physical exercise and nutrition interventions in adult patients before elective major surgery.

**Recent Findings:**

Exercise training before elective adult major surgery is feasible, safe, and efficacious, but the clinical effectiveness remains uncertain. Early data suggests a reduction in morbidity, length of stay, and quality of life, but the results of larger definitive studies are awaited. Nutritional interventions are less well evaluated and when they are, it is often in combination with exercise interventions as part of a prehabilitation package.

**Summary:**

Studies evaluating exercise and nutrition interventions before elective major surgery in adults are producing encouraging early results, but definitive clinical evidence is currently very limited. Future research should focus on refining interventions, exploring mechanism, and evaluating the interactions between therapies and large-scale clinical effectiveness studies.

## Introduction

Surgical trauma, the physiological consequences of anesthesia, perioperative therapies (including fluids and oxygen) along with psychological distress are major stressors that patients face in the perioperative period. Resilience to these stressors is dependent on a number of factors including age, chronic health status, and acute physiological derangements consequent upon the presenting illness. Additionally, a number of factors directly related to long-term behaviors are key determinants of perioperative physiological resilience. These include physical activity/exercise, nutritional status, smoking, and alcohol consumption. In turn, these factors interact with disease-related factors including cancer cachexia, malabsorption, and myopathy. Prehabilitation is the process of enhancing the functional capacity of an individual to enable him or her to withstand a stressful event [1], in this case, in the context of surgery.

Surgery, despite its inherited physiologic stress, remains a cornerstone of modern medicine. Minimally invasive and robotic approaches are significant recent innovations, and enhanced recovery pathways have become standard of care in the majority of centers for all surgical patients. Combined, these novel approaches have made surgery safer, with a significantly lower stress response and improved clinical and oncological outcomes. Despite these innovations, major cancer surgery, in particular, still carries a substantial mortality [2, 3] and high morbidity [4••]. Postoperative complications prolong length of hospital stay, increase costs, increase readmissions, and impair “back to baseline” recovery. Outcomes from surgery have far reaching effects that may not be appreciated by perioperative health professionals, including an impaired ability to return to a pre-illness level of activity, fitness, and quality of life. This persistent postoperative impairment is especially apparent in the frail and elderly and may be one of the main mechanisms underpinning the recognized relationship between short-term complications and long-term survival following surgery [5, 6].

Patients arriving at the doorstep of major elective surgery are rarely offered a perioperative optimization package, mainly due to surgical pathways designed with different aims in mind and rigid time constraints between the time surgery is first contemplated and the actual surgical date, especially for cancer patients. Opportunities to optimize modifiable risk factors such as pre-existing comorbidity, fitness, nutrition, psychology, and deleterious effects from neoadjuvant cancer therapies are being missed [7]. Patients are likely to be particularly amenable to interventions that positively change behavior when faced with an impending major life event, such as major surgery, and this may offer a particular “teachable moment.” Given this likely highly motivated patient population, health professionals are in a unique position to deliver tailor-made interventions in order to instigate a positive influence on perioperative outcomes. Multimodal surgical prehabilitation including exercise to improve physical fitness, nutrition optimization, smoking/alcohol cessation, and psychological stress reduction interventions are gaining traction worldwide. Routine preoperative optimization using a multimodal prehabilitation approach has the potential to benefit a large number of patients through benefits on short-term clinical outcome as well as offering the possibility of long-term behavioral change and health benefits. This radical shift in ethos will in many cases require radical re-design of the “surgical pathway” so that perioperative physicians interact with patients earlier in the perioperative journey, opening up numerous opportunities to improve patient care. Such a change will also offer enhanced collaborative decision-making ensuring that patients make the most informed decision about which treatment option they wish to pursue, including whether to have an operation or not, well before the day of surgery. Pathway re-design and prehabilitation offer a route to improving modifiable behavioral characteristics prior to surgery through active programs of alcohol cessation, smoking cessation, activity/exercise, and dietary intervention. The advent of “Surgery Schools” gives us the opportunity to share such knowledge with our patients and thereby guide them toward healthier behaviors. Surgery schools also offer the opportunity to manage expectations in relation to the in-hospital surgical journey, improve buy-in to enhanced recovery pathways, and improve psychological preparation for surgery. Together, this multimodal package offers an opportunity for a multitude of gains to be made prior to surgical interventions with the aim of maximizing our patients’ resilience to the physiological and psychological stresses of surgery through targeted management of modifiable risk factors.

This chapter seeks to discuss topical points around multimodal prehabilitation strategies particularly focusing on exercise and nutrition interventions, with the aim of evaluating their impact on perioperative outcomes.

### Exercise Prehabilitation

Lack of physical activity is one of the major modifiable risk factors of ill-health [8] and premature death, along with poor nutrition, smoking, and alcohol [9]. We live in a sedentary society in which we habitually drive cars instead of walking or cycling, sit for long periods in front of computer monitors and televisions, and build environments in which exercise and activity are minimized. Nevertheless, there is a large body of evidence supporting the notion that physical fitness has benefits in almost every context of health and disease, advocating better outcomes for fitter people [10]. Furthermore, that physical *inactivity* is one of the leading public health issues we face [11, 12]. A decline in physical activity as a result of aging or critical illness results in a significant increase in perioperative risk that may be attenuated by physical exercise interventions.

The link between physical activity and cancer risk is quite clear. The largest review [13•] was a pooled analysis of 12 prospective European and US cohorts that included 1.44 million participants and 186,932 cases of cancer with self-reported physical activity. This pooled analysis concluded that high levels of physical activity during leisure time (the 90th percentile compared with the 10th percentile) were associated with reduced risks of 13 types of cancer. A review [14] of 126 studies found a 10% reduction in risk across cancer types associated with physical activity, but a threshold effect meant that physical activity exceeding two times the current recommendations did not provide additional benefits. Increasing patient physical activity is therefore believed to reduce the risk of developing cancers because of its role in helping to maintain a healthy weight, although activity has numerous other beneficial effects on health and disease risk. The biological bases underlying the associations between physical activity and cancer risk are incompletely defined [15•].

Interventions to improve post-surgical recovery have usually been targeted at the intra-operative and postoperative periods. For high-risk patients about to undergo major surgery however, this is likely to be too late. Poor objectively measured physical fitness is linked to poor postoperative outcomes [16••]; therefore, identifying interventions to optimize preoperative fitness prior to major surgery is a priority. As discussed, the preoperative period may also be an emotionally salient time to engage patients in enhancing their physical fitness prior to embarking on their surgical journey. Prehabilitation is defined as “the process of enhancing the functional capacity/ physical fitness of an individual to enable them to withstand a stressful event” [1]. Physical exercise training prior to elective surgery meets this criterion.

Aerobic and muscular strength training in major surgical patients has been shown to increase endurance, improve objective markers of physical fitness, reduce weight gain, and improve muscle strength. Although constraints to proceeding with surgery limit the time for the initiation of prehabilitation, a *3-week period* may still be sufficient to obtain a moderate gain in aerobic and muscle strength reserve. Importantly, in neoadjuvant cancer therapies, which are typically administered prior to surgery and followed by a recovery period of 6 to 12 weeks (or more), have opened up a time window to train patients prior to major cancer operations where previously the pressure of reducing the time between diagnosis and surgery precluded such an intervention. A critical aspect of improving physiological reserve lies in the ability to cope with surgical trauma/stress. Although a decrease in functional capacity in the period after surgery is recognized, primarily caused by surgical trauma, inflammation, or the cancer itself [1], this is further amplified by a reduced innate patient reserve. Bed rest, the need to “take it easy,” and inactivity due to cancer treatments all compound the poor outcomes we observe postoperatively.

Early studies on prehabilitation before major thoracic and abdominal surgery have shown an increase in preoperative physical fitness, physical activity, and shorter hospital stay [17–20]. Feasibility and safety, even after neoadjuvant cancer treatments, as well as improvements in physical activity and quality of life [20, 21], have also been demonstrated. Reviews on pre-surgical exercise training in patients undergoing cancer treatments in both adjuvant and neoadjuvant periods also show a reassuring improvement in fitness, however again fall short on clearly identifying a postoperative outcome benefit [22•, 23•, 24•]. In the surgical oncology setting (adjuvant), only one study in breast cancer patients showed significant improvements in physical fitness after a 16-week exercise training program. Yet other exercise training studies showed improvements in other important outcomes such as quality of life and fatigue. In people with newly diagnosed cancer (neoadjuvant setting), three pilot studies showed clinical and significant improvements in objectively measured fitness variables after a supervised in-hospital interval training in people with rectal cancer [19] and breast cancer [25]. More recently, interest has shifted toward designing high-intensity training (HIT) programs in the preoperative setting, which may allow for effective and time-efficient exercise training before surgery [26]. Supervised HIT programs, carefully designed and individually tailored, targeting the upper and the lower body, may be a valuable addition to the perioperative pathway. However, to date, no published study has investigated HIT in people with cancer.

More recently, several systematic reviews [27, 28, 29••] have reviewed exercise prehabilitation in abdominal cancer surgery especially in colorectal surgery. They all agree that exercise prehabilitation is a possible means of enhancing physical fitness and quality of life; however, no clear impact on postoperative outcomes is currently acknowledged. Moran and colleagues [29••] reviewed studies conducting prehabilitation which consisted of inspiratory muscle training, aerobic, and resistance exercise training. These training modalities appear to decrease the incidence of postoperative complications in patients undergoing intra-abdominal surgery. However, this effect was strongest when prehabilitation was compared with usual care or breathing exercises only (OR 0.35, 95% CI 0.17–0.71). Furthermore, prehabilitation significantly decreased the incidence of postoperative pulmonary complications (OR 0.27, 95% CI 0.13–0.57), which were measured as the primary complication of interest in the majority of studies reviewed. The potential for interventions that achieve maximum results over short periods needs to be urgently explored. A recent meta-analysis concluded that interval training was more effective than continuous training at increasing fitness and demonstrated a similar safety profile for moderate-intensity training [30]. A preoperative, supervised, high-intensity program of interval training may increase a patient’s aerobic capacity prior to an operation within a short time frame [26]; however, an easier alternative is a walking-based intervention, which can be performed by patients at home. However, this type of moderate-intensity exercise may not create the improvements necessary within a short time frame. The ability of these programs to improve aerobic fitness should be compared in future research.

Encouragingly, this area of research is filled with new studies attempting to answer specific questions relating to longer term outcomes, e.g., CHALLENGE study (Colon Health and Life-Long Exercise Change) [31], the INTERVAL-MCRPC study (Intense Exercise for Survival among men with Metastatic Castrate-Resistant Prostate Cancer), and the PANTERA study (Exercise as Treatment for Men with Prostate Cancer) and started enrolment in 2016. PREPARE ABC (SupPoRtive Exercise Programmes for Accelerating REcovery after major ABdominal Cancer surgery), PREHAB (multimodal prehabilitation in colorectal cancer patients to improve functional capacity and reduce postoperative complications), and WesFIT (a pragmatic parallel group design randomized controlled study to assess the efficacy of the implementation of a prehabilitation program in patients undergoing elective major cancer surgery in Wessex, UK) trials will start recruiting later part of 2017. An improved understanding of the optimal training duration, pattern, intensity, and composition of such interventions will be needed to maximize efficacy. Furthermore, in order to maximize the effectiveness of training, a better understanding of the complex interplay between adherence, efficacy, “responders versus non-responders” to exercise, and cost for in-hospital supervised training interventions versus self-directed outpatient approaches is also urgently needed. The impact of multimodal prehabilitation and its impact on traditional surgical outcomes like morbidity, overall survival, and oncological outcomes, as well as the mechanisms driving these changes in physiology, biology, and possibly cancer biology with tailored exercise, also need exploring.

### Nutritional Prehabilitation

Malnutrition arises from inadequate intake and/or metabolic and inflammatory alterations that alter nutrient utilization (hypermetabolism/catabolism), requirement or absorption, which ultimately leads to wasting, cachexia, decreased physical fitness, and reduced metabolic reserve. The primary goals of nutritional prehabilitation are to optimize nutrient stores and metabolic reserve preoperatively and provide an adequate buffer to compensate for the catabolic response of critical illness or surgery. Nutritional prehabilitation is different from acutely replacing nutritional deficits. To be successful, nutritional intervention requires a timeline that needs to start at contemplation of surgery to ensure early patient engagement [32•] and must extend into the perioperative and postoperative periods. The shift to pre-emptive rather than reactive nutrition assessments and intervention must be emphasized. The recent European Society for Clinical Nutrition and Metabolism (ESPEN) guidelines [33••] clearly show the prognostic influence of nutritional status on complications and mortality. Malnourished surgical patients have significantly higher postoperative morbidity, mortality, length of hospital stay, readmission rates, and increased costs associated with their inpatient episodes [9, 34, 35]. This risk of malnutrition is often most significant prior to and following major gastrointestinal (GI) and cancer surgery [9, 36, 37] which also often demonstrate the greatest risk of iatrogenic and baseline malnutrition (~ 65%) [38]. It is essential that the chronically malnourished cachectic cancer patient is identified via early preoperative assessment and that they receive adequate nutritional intervention prior to major surgery [32•, 37], even if it may mean a brief delay in operation time to optimize nutrition status first. Appropriate perioperative nutritional interventions have been shown to specifically improve perioperative outcomes in GI and cancer surgical patients [38], specifically reducing surgical site infections [9, 33••, 34, 35]. There is a long history of randomized controlled trials (RCTs) and meta-analyses demonstrating that preoperative nutrition (regardless of route of administration) in malnourished patients prior to GI surgery reduces postoperative morbidity by 20% [39]. Unfortunately, the success of surgery does not depend exclusively on technical surgical skills, but also on how patients respond to surgical and physiological stressors; hence, delivering nutrition, ideally preoperatively and not reactively in the perioperative period, is of utmost importance. The major effect of surgery and critical illness to induce protein catabolism also needs to be understood and emphasized. Provisions of protein, independent of whether energy or total calorie requirements, are met, can maintain lean muscle mass, and reduce the risk of subsequent frailty in the elderly [40, 41]. Finally, a recent trial conducted in colorectal surgery patients within an enhanced recover pathway demonstrated that patients receiving high protein oral nutrition supplements postoperatively (consumption of > 60% of protein needs over first 3 postoperative days) was associated with a 4.4-day reduction in length of stay (*p* < 0.001) [42••].

Cancer cachexia is prevalent in 50–80% of the people with cancer [43]. Cancer cachexia due to tumor-induced anorexia, catabolic effects of the tumor, abnormal metabolism of nutrients, physical obstruction of the gastrointestinal tract, reduced food intake after cancer treatment, and diminished intake due to pain, anxiety, and depression must be recognized, assessed, and intervened upon preoperatively. Traditionally, body weight and body mass index (BMI) have been used as surrogate measures of nutritional status, with large studies in patients with lung, GI, and other cancers illustrating a strong relationship between weight loss or low muscle mass and survival, i.e., people with cancer who lost more weight had reduced survival compared with those who did not lose as much weight [44]. Specifically, patients with significant weight loss and sarcopenia due to their cancer survived an average of 8 versus 28 months in patients without weight loss/sarcopenia. Body weight and BMI still remain important components of nutritional assessment, with more recent studies validating a BMI-adjusted weight loss grading system [45]. The Subjective Global Assessment (SGA) and the Patient-Generated Subjective Global Assessment (PG-SGA) screening tools are the most documented tools in the oncology literature [46•]. Numerous other screening tools have been validated for use on hospitalized patients, including the Malnutrition Universal Screening Tool (MUST), the Malnutrition Screening Tool (MST), Nutrition Risk Screening 2002 (NRS-2002), and the Short Nutrition Assessment Questionnaire (SNAQ©). A total of 32 nutritional risk assessment tools exist, which were identified in 83 studies after a systematic review of the literature [47••]. Although many nutrition screening tools do exist, there is no consensus related to the optimal screening tool for use in the preoperative “at risk” surgical patient population as all the above screening tools are intended for hospitalized patients.

Unfortunately, recent evidence reveals significant deficiencies in nutritional screening and intervention in US and European colorectal and oncologic surgical patients with only ~ 1 in five US hospitals currently utilizing a formal nutrition screening process [48•]. This is surprising as 83% of US surgeons believe existing data supports preoperative nutrition optimization to reduce perioperative complications. However, only 20% of US GI/oncologic surgery patients receive any nutritional supplements in the preoperative or postoperative setting. Overall, US surgeons recognized both importance of proper perioperative surgical nutritional support and the potential value to patient outcomes.

#### Perioperative Nutrition Screening—Proposal for a Novel Screening Tool: PONS Score

**Fig. 1:**
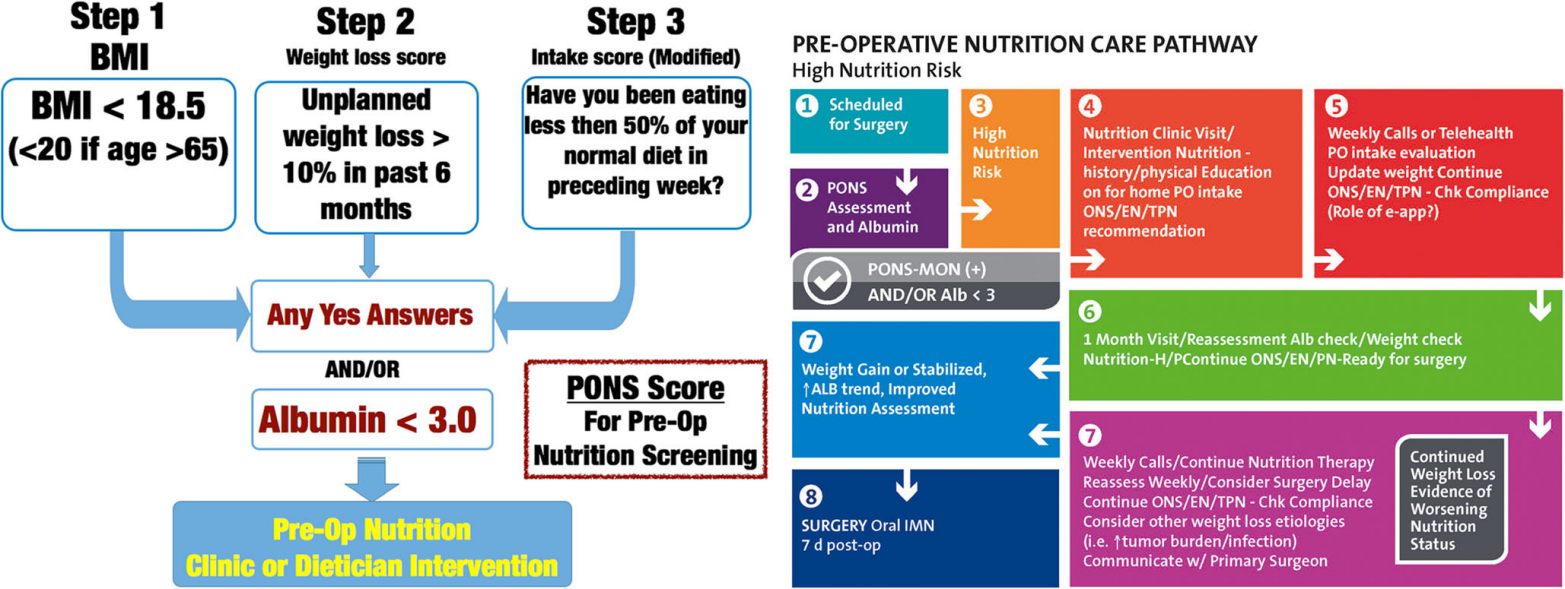
Preoperative nutrition score (PONS) assessment tool with an example of a preoperative nutritional care pathway for high nutrition risk patients—as defined by any positive response on the PONS score. (Currently utilized by Duke University Perioperative Optimization Team (POET) Nutrition Clinic). POQI copyright adapted from reference [49]

Recently, our Perioperative Quality Initiative (POQI) group [49••] conducted an extensive literature review and developed and proposed a novel perioperative nutrition screen—the PONS score (perioperative nutrition screen (PONS)). As shown in Fig. 1, the PONS is a modified version of the Malnutrition Universal Screening Tool (MUST) [50] that has been modified for perioperative use. The PONS determines the presence of nutrition risk based on a patient’s BMI, recent changes in weight, reported recent decrease in dietary intake, and preoperative albumin level. In addition, the PONS includes evaluation of preoperative albumin level, as this is a predictor of postoperative complications, including morbidity/mortality [50, 51•, 52–54].

The PONS can be easily administered and incorporated into an electronic medical record for efficient communication. The intent is that the PONS can be administered quickly (< 5 min) by nursing staff in surgical/preoperative clinics, and the results will be instantly uploaded into EMR, automatically triggering a nutrition intervention if one or more positive responses on the PONS score are recorded. Patients who are identified as being at high nutrition risk upon screening should be referred to a registered dietitian nutritionist (RDN) for a complete nutrition assessment and intervention. In situations where referrals to RDNs are not possible, oral nutritional supplements (ONS) or other appropriate nutrition intervention is recommended for a period of approximately 4 weeks prior to surgery to optimize nutrition status as described in POQI High Nutrition Risk Pathway (Fig. 1).

#### Perioperative Nutrition Intervention

A recent Cochrane review analyzing evidence of preoperative nutritional interventions on postoperative outcomes and length of hospital stay with the use of standard preoperative oral nutritional supplements in patients undergoing GI surgery was undertaken [55]. This identified a significant reduction in total postoperative complications with immune and parenteral nutrition, in predominantly malnourished participants. Two trials evaluating enteral nutrition and three trials evaluating standard oral supplements were also included, neither of which showed any difference in the primary outcomes. Specific to high nutrition risk perioperative cancer patients, the incidence of surgical site infections was significantly lower in the group receiving adequate energy support via oral, enteral nutrition (EN), and/ or parenteral nutrition (PN) for at least 10 days than in group with inadequate/no support for < 10 days (17.0 vs. 45.4%, *p* = 0.00069). In multivariate analysis, nutritional therapy was an independent factor associated with fewer surgical site infections (OR 0.14, *p* = 0.0002) [56••]. When oral nutrition is unable to meet the protein and calorie requirements in malnourished patients, enteral supplementation should be preferred over PN whenever possible. A period of 7–14 days of PN is recommended. If PN is required to meet energy needs, it should be combined whenever possible with EN or ONS. For surgical patients, the benefits of nutritional therapy have been consistently shown in cases of severe under nutrition [57–59] and further confirmed by two meta-analyses [60, 61].

Specific to the benefits of ONS in patients undergoing major surgery requiring hospitalization, a large body of data demonstrates benefits of high-protein ONS. Meta-analysis data in a range of hospitalized patients, including surgery, demonstrates ONS reduces mortality, reduces hospital complications, reduces hospital readmissions, shorten length of stay, and reduces hospital costs [62, 63••, 64]. A large hospital database analysis of ONS use, in 724,000 patients matched with controls not receiving ONS, showed a 21% reduction in hospital LOS and for every $1 (US) spent on ONS, $52.63 was saved in hospital costs [65]. Further research focused on the high-risk perioperative patients is needed to optimize perioperative nutrition delivery.

### Combinations of Nutrition and Exercise to Optimize Perioperative Outcomes

A combination of both individualized nutrition counseling, oral nutritional supplements, and exercise has been proven to be effective in building physical fitness in prehabilitation trials [66, 67•, 68–70]. In 2013, Denison and colleagues [71•] conducted a systematic review including 17 randomized controlled trials (RCTs) to explore the effect of combined exercise and nutrition intervention to improve muscle mass, muscle strength (measures of sarcopenia), and physical performance in older people (all over 60 years old). They concluded that further studies were needed to provide evidence upon which public health and clinical recommendations could be based. A recently updated systematic review from the same group [72••] was published identifying 21 additional RCTs (total of 37 RCTs). In 79% of the studies (27/34 RCTs), muscle mass increased with exercise but an additional effect of nutrition was only found in eight RCTs (23.5%). Muscle strength increased in 82.8% of the studies (29/35 RCTs) following exercise intervention, and dietary supplementation showed additional benefits in only a small number of studies (8/35 RCTS, 22.8%). The majority of studies showed an increase of physical performance following exercise intervention (26/28 RCTs, 92.8%), but interaction with nutrition supplementation was only found in 14.3% of these studies (4/28 RCTs). The review concludes that physical exercise has a positive impact on muscle mass and muscle function in healthy subjects aged 60 years and older with a large effect seen with exercise interventions of any type. However, a large variation in regard to the dietary interventions was found, and this is likely to be essential to potential benefit, especially with regard to protein content, type, supplemental nutrient delivery, and overall total caloric delivery. Moreover, using the selected inclusion criteria, the studies, this review captured, predominantly included well-nourished elderly subjects; hence, its translatability to a malnourished surgical population is very limited.

Very few well-designed multimodal exercise and nutrition prehabilitation studies have been undertaken. Gillis and colleagues [68] undertook a parallel-arm single-blind superiority randomized controlled of 70 colorectal cancer patients who were randomized to receive either prehabilitation (*n* = 38) or rehabilitation (*n* = 39). The prehabilitation group increased their functional walking capacity by ≥ 20 m compared with the rehabilitation alone group (53 vs. 15%, adjusted *p* = 0.006). Complication rates and duration of hospital stay between the two groups were similar. Another study published by the same group [67•] undertook a study to interrogate the impact of nutrition counseling and whey protein supplementation on preoperative functional walking capacity and recovery in patients undergoing colorectal resection for cancer. A double-blinded randomized controlled trial in 48 patients scheduled for elective colon cancer surgery was randomized to receive either individualized nutrition counseling with whey protein supplementation to meet protein needs or individualized nutrition counseling with a nonnutritive placebo. Counseling and supplementation began 4 weeks before surgery and continued for 4 weeks after surgery. Clinically meaningful improvements in functional walking capacity were achieved before surgery with whey protein supplementation. Burden [69] evaluated the effect of preoperative standardized oral supplements in a cohort of colorectal cancer patients. In a randomized controlled trial, patients were assigned to receive 400 mL of oral supplement and dietary advice or dietary advice alone. The intention-to-treat analysis identified no statistically significant difference between the intervention and control groups for the primary outcome (i.e., total postoperative complications). The results of the ongoing multimodal prehabilitation exercise and nutrition studies, with clinically relevant endpoints, will be reported in the next few years and inform our management.

### Future Direction

Substantial gains to improve surgical outcomes via optimization, utilizing multimodal prehabilitation intervention, are yet to be made. We know that poor preoperative physical fitness reflecting poor physiological reserves is associated with postoperative morbidity, and that prehabilitation prior to acute or chronic stressors can improve fitness and quality of life. A number of promising opportunities are being developed in perioperative medicine, including increasingly sophisticated risk prediction, collaborative decision-making, personalized medicine, and targeted multimodal interventions. It therefore seems reasonable to enhance our current preoperative assessments by incorporating nutritional and objective preoperative physical fitness risk screening that is intuitively easy to comprehend. The idea of “fitness for surgery” is essential for discussions about the specific risks and benefits of a particular procedure for a particular patient. Personalized medicine, via prescribing tailored exercise and nutritional interventions, aimed at improving surgical outcomes may be used to guide operative interventions, postoperative care, cancer therapies (including the selection of chemotherapy and timing of cancer treatments in relation to surgery), and choices of appropriate multimodal prehabilitation/rehabilitation programs. Mechanisms underpinning the interactions of changes in fitness with changes in tumor microenvironment, cancer therapies, and exercise are largely unknown, so work within this area is urgently needed.

## Conclusions

Improving resilience to the physiological stresses of surgery, anesthesia, and the perioperative course is an attractive approach to improving outcome following surgery. Physical exercise and nutritional therapies are important candidates in this role. Exercise training before adult major surgery is feasible, safe, and efficacious, but the clinical effectiveness remains uncertain. Early data suggests a reduction in morbidity, length of stay, and improvement in quality of life, but the results of larger definitive studies are awaited. Nutritional interventions are less well evaluated and when they are, it is often in combination with exercise interventions, as part of a prehabilitation package. These encouraging early results merit further evaluation that should focus on refining interventions, exploring mechanism, and evaluating the interactions between therapies and large-scale clinical effectiveness studies.
